# Rare Variant of Left Circumflex Coronary Artery Originating From the Right Coronary Artery

**DOI:** 10.7759/cureus.27265

**Published:** 2022-07-25

**Authors:** Abdalhai Alshoubi, Brian Kurtz, Alan Dean, Adam Willey, Erika Keshishian

**Affiliations:** 1 Clinical Anesthesiology and Critical Care Medicine, St. Joseph’s Medical Center, Stockton, USA; 2 Anesthesiology and Critical Care, St. Joseph’s Medical Center, Stockton, USA; 3 Anesthesiology and Critical Care, St. Joseph's Medical Center, Stockton, USA

**Keywords:** myocardial infarction, coronary artery disease, coronary angiography, anomalous origin, variant of left circumflex artery

## Abstract

Coronary artery anomalies are rare in the general population. Many individuals with coronary artery anomalies are asymptomatic. Some individuals with these anomalies have an increased risk of sudden cardiac death (SCD), especially young athletes, and an elevated risk of myocardial ischemia with anginal symptoms, seen in older age groups.

We report a 43-year-old male who received coronary artery bypass graft (CABG) surgery for the four-vessel disease after suffering from an anteroseptal myocardial infarction (MI). The patient presented to the hospital emergency department with episodes of chest pain for three days. On coronary angiography, an anomalous origin of the left circumflex coronary artery (ALCx) was visualized. This ALCx was a type I variant originating from a separate ostium from the right coronary artery (RCA) at the right coronary cusp.

It is important to document and describe the different variants of coronary anomalies to provide proper patient management. The anomalous origin of the left circumflex coronary artery from the right coronary cusp of the RCA is considered a benign variant. It may, however, have been instrumental in supplying blood to the left heart in the setting of complete left coronary artery (LCA) occlusion.

## Introduction

Congenital coronary anomalies have a relative rate of occurrence of 0.2%-1.3% based on adult angiographic series [1]. With a rate of 0.37%-0.7%, the left circumflex coronary artery (ALCx) is the most common coronary anatomic variation [2,3]. Typical origins of the ALCx include the right sinus of Valsalva or proximal branching off the right coronary artery (RCA) [4]. Three different types of ALCx have been documented: Type I (separate Ostia for RCA and LCx), Type II (common Ostia in the right sinus), and Type III (LCx arising as a branch of the proximal RCA) [5]. Some cases have no clinical significance, while others have been known to cause non-atherosclerotic coronary artery disease. There have been cases of ALCx originating from the right aortic sinus with clinical presentations of myocardial infarction or effort angina that have no atherosclerotic lesions [6]. 

We report a rare variant of the LCx artery originating from the right coronary cusp close to the origin of the RCA in a patient presenting with acute anteroseptal ST-elevation myocardial infarction.

## Case presentation

**Figure 1:**
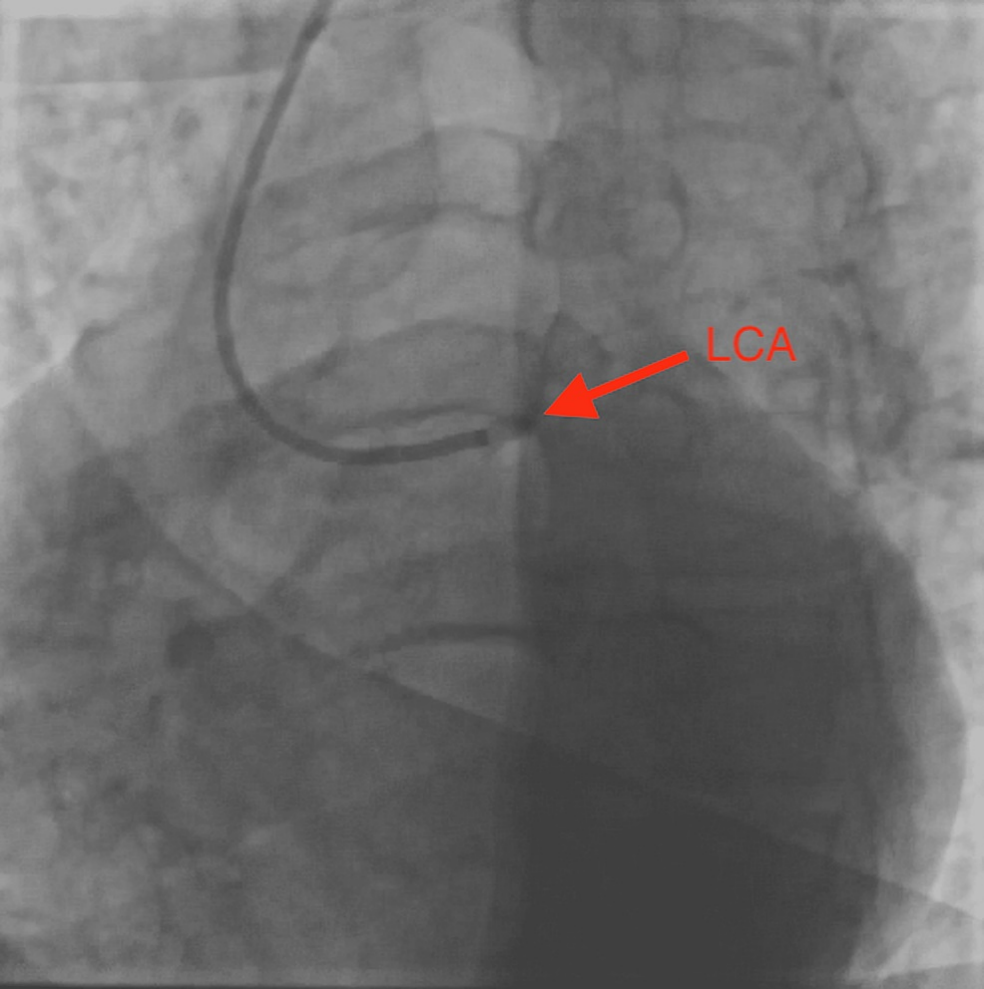
Coronary angiography showing complete occlusion of the left coronary artery (LCA)

**Figure 2:**
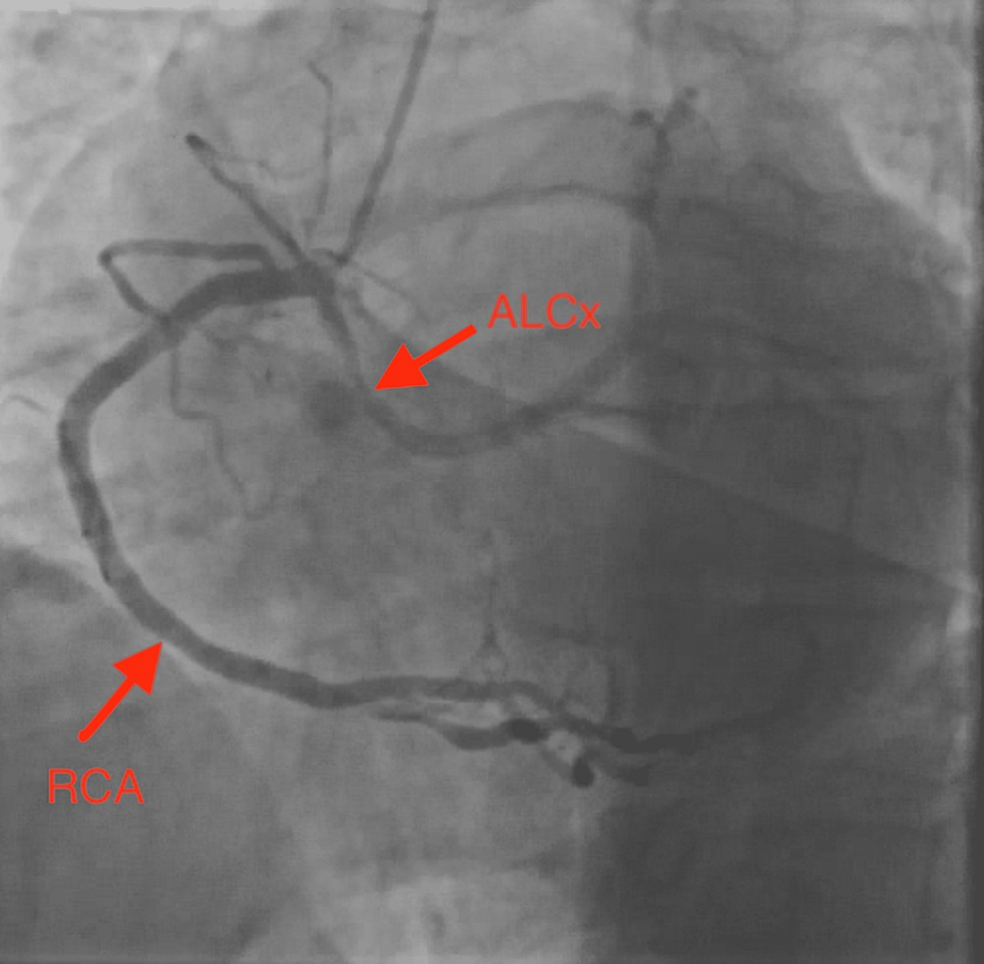
Anomalous left circumflex artery originating from right coronary artery angiography. ALCx = anomalous left circumflex artery, RCA= right coronary artery

A 43-year-old gentleman with a significant history of diabetes, hypertension, hyperlipidemia, obesity, smoking, and a family history of coronary artery disease, presented to the hospital emergency room with the complaints of having a few days of chest pain, radiating to the back as well as to the left arm. His heart rate was 73 beats per minute, blood pressure 150/92 mmHg, respiratory rate 13 cycles per minute, and oxygen saturation 98% on room air. In the emergency room, he was ruled in for acute anteroseptal ST-elevation myocardial infarction, possibly with a delayed presentation as his chest pain has been going on for three days, and deep Q waves were seen in the septal leads. The patient underwent heart catheterization, which showed complete occlusion of the left main coronary artery (Figure 1), and 90% stenosis at the bifurcation between the left anterior descending and diagonal arteries anteriorly. Concerning the posterior blood supply, the right coronary artery was dominant, and the branching posterior descending artery showed 80% stenosis. Catheterization also revealed an anomalous origin of the left circumflex coronary artery from the right coronary cusp close to the origin of the right coronary artery (Figure 2).

This was 70% stenosed in its proximal section. The patient was hemodynamically unstable with a blood pressure of 89/64 mmHg due to the anteroseptal ST-elevation myocardial infarction. Left ventriculography showed an ejection fraction of 35%-40% with severe hypokinesia of the anterior wall. The patient's vital signs stabilized after the placement of an intra-aortic balloon pump and starting vasopressors. 

**Figure 3:**
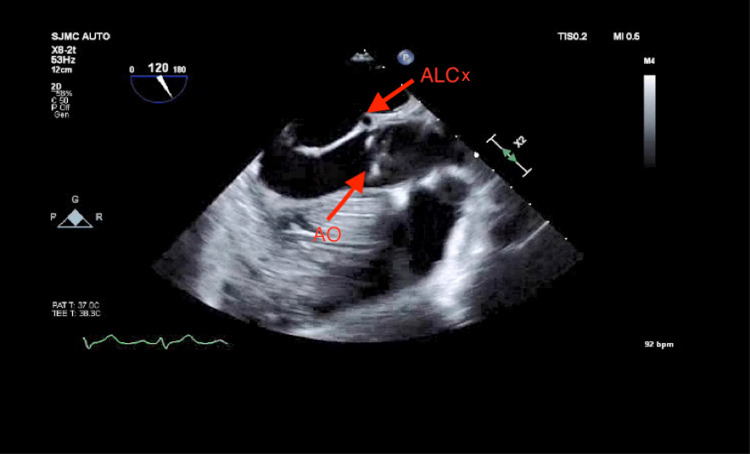
Anomalous left circumflex artery originating from right coronary artery transesophageal echocardiogram, long-axis view, AO= aortic valve, ALCX = anomalous left circumflex artery

The patient underwent emergent coronary artery quadruple bypass graft surgery. The left internal mammary artery was harvested and anastomosed to the left anterior descending artery. The saphenous vein was harvested and used for three separate grafts; it was used in anastomoses to the posterior descending artery, the obtuse marginal-1 artery, and the diagonal artery. The mid-esophageal long-axis view of transesophageal echocardiography (TEE) showed a single coronary artery originating from the right coronary sinus, which becomes ALCx (Figure 3).

**Figure 4:**
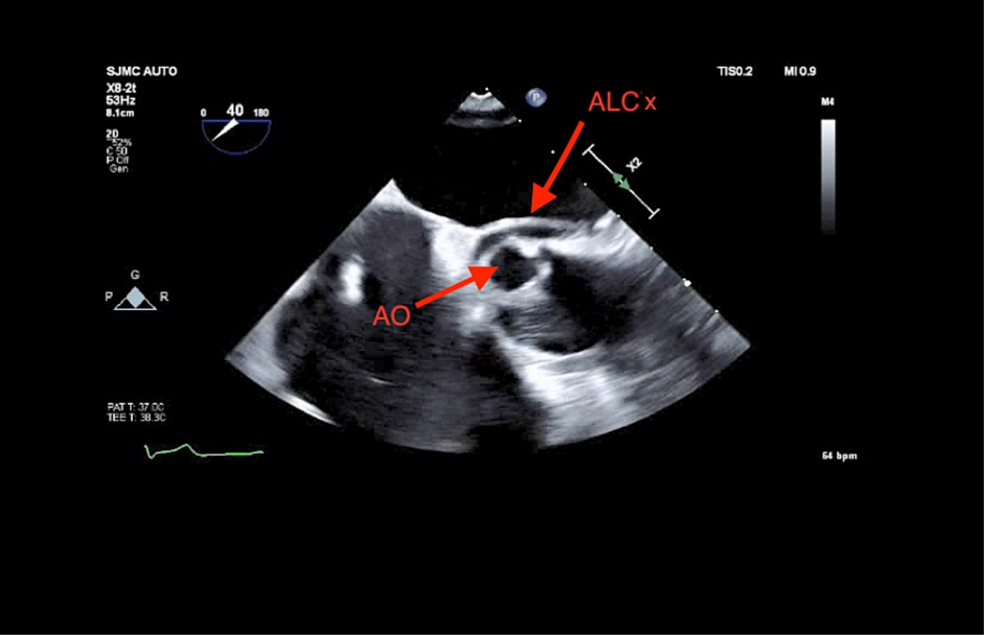
Anomalous left circumflex artery originating from right coronary artery transesophageal echocardiogram, short-axis view, AO= aortic valve, ALCx = anomalous left circumflex artery

The ALCx also can be seen in the mid-esophageal short-axis view of TEE (Figure 4).

Postoperatively, the patient required vasopressors and an intra-aortic balloon pump which was successfully weaned on a postoperative day one. The patient made full recovery and was discharged home six days later.

## Discussion

Variant LCx arteries arising from the right sinus of Valsalva or RCA are one of the more common variations observed in coronary anatomy [7]. In a Cleveland Clinic study that compiled data for nearly 30 years of arteriography and examined 126,595 U.S. patients, it was reported to have a prevalence of 0.37% [7]. Other studies have found a prevalence of 0.67% in 2,996 U.S. patients and 0.17% in 16,573 patients from Turkey [3]. Many anomalous coronary variations exist, but others that have been more heavily studied include a high origin of a coronary artery, where the artery arises above the sinotubular junction on the aorta, a left main coronary artery (LCA) branching from the proximal RCA, or the RCA branching from the proximal left coronary artery (LCA) [8]. These anomalies have been implicated in sudden cardiac death (SCD), with the LCA branching from the proximal region of the RCA showing the greatest incidence [9-11]. 

Attention has focused on the mechanism behind SCD with these coronary anatomic variants, and it appears to be myocardial ischemia related to arterial compression or structural variations that limit blood supply. These include Ostia that are narrowed or irregular, acute angle take-off, where the anomalous artery branches sharply from its parent vessel, and arterial course [12]. The anomalous arteries may move between the aorta and pulmonary artery interarterially and occasionally arise intramurally within the aortic wall [8,13]. Any coronary anomaly that decreases myocardial perfusion can put a patient at risk for SCD. When the LCx arises from the right coronary sinus, it typically moves in a retro aortic manner, posteriorly looping around the aorta towards the left lateral wall of the heart to supply the left myocardium [7,8]. With this course, it does not cross interarterially and is typically considered a benign variant [7,13]. Some case reports have described adverse events such as myocardial ischemia believed to be related to acute angle take-off or atypical chest pain without other evidence of compression or luminal narrowing [2,5]. Another describes SCD in a patient who was subsequently found to have no evidence of obstructive atherosclerosis but did have an anomalous LCx artery branching from the right sinus of Valsalva [14]. Others describe the variant as an incidental finding after undergoing angiography for another indication [4,15,16]. Although individual case reports have indicated adverse events, large-scale studies examining this variant did not report an increased incidence of SCD, angina, atherosclerosis, or myocardial infarction, and all have described it as benign [7,9,17]. Clinically, the ALCx branching from the proximal RCA is important to note during cardiac surgery. There have been multiple cases of LCx compression with this variant, sometimes resulting in infarction. All incidents have occurred during valve replacement surgery [18-20]. 

In the case of our patient, the anomalous left circumflex artery arising from the right sinus may have been crucial for his survival. As his LCA was completely occluded, and LCx artery originating from the LCA would not have received adequate blood flow, and his anteroseptal infarction would have spread over a much greater territory. His LCx artery was one of the least stenosed of his major vessels and likely supplied a critical amount of myocardium.

## Conclusions

Congenital coronary artery anomalies are rare. The most common type of ALCx is type I, where ALCx originates from a separate ostium of the right coronary artery. In our report, we present a patient who suffered from a diffuse atherosclerotic disease of his coronary arteries and ALCx type I. This variant is considered benign; however, recognizing the presence of this anomaly is crucial, especially in valve surgery, as this artery might get compressed. The presence of this anomaly in our patient may have been instrumental in supplying blood to the left heart when his LCA was completely occluded.
